# Supplementary material for: Lifecourse body mass index trajectories and cardio-metabolic disease risk in Guatemalan adults
Source: PLoS One. 2020 Oct 22;15(10):e0240904. doi: 10.1371/journal.pone.0240904 (PMC7580923; doi:10.1371/journal.pone.0240904)
Supplement: S1 Table — (DOCX) [file pone.0240904.s001.docx]

S1 Table. Effect sizes for BMI in 2015-17 After 40 Years of Follow Up at Age 37-54 Years From Multivariable Logistic Regression Models Predicting Cardio-metabolic Disease Risk Factors in 2015-17 in the INCAP Nutrition Supplementation Trial Longitudinal Cohort (n=510 Women, n=346 Men).

|  | Women | | Men | |
| --- | --- | --- | --- | --- |
|  | BMI in 2015-17  (1 kg/m^2^ units),  centered at 25 kg/m^2^ | | BMI in 2015-17  (1 kg/m^2^ units),  centered at 25 kg/m^2^ | |
| Cardio-metabolic risk factor | OR (95% CI) | *P* | OR (95% CI) | *P* |
| Abdominal obesity defined by waist circumference ^a^ | 3.00 (2.16, 4.18) ^b^ | <0.0001 | 4.63 (2.95, 7.28) | <0.0001 |
|  |  |  |  |  |
| Obesity defined by % body fat ^c^ | 1.65 (1.34, 2.04) | <0.0001 | 1.55 (1.33, 1.81) | <0.0001 |
|  |  |  |  |  |
| Elevated triglycerides ^d^ | 1.07 (1.01, 1.13) | 0.01 | 1.21 (1.10, 1.32) | <0.0001 |
|  |  |  |  |  |
| Low HDL-c ^e^ | 1.10 (1.03, 1.18) ^b^ | 0.006 | 1.25 (1.13, 1.39) | <0.0001 |
|  |  |  |  |  |
| Diabetes ^f^ | 1.00 (0.96, 1.05) | 0.9 | 1.00 (0.90, 1.11) | 0.9 |
|  |  |  |  |  |
| Hypertension ^g^ | 1.10 (1.06, 1.15) | <0.0001 | 1.15 (1.07, 1.24) | 0.0002 |
|  |  |  |  |  |
| Metabolic syndrome ^h^ | 1.22 (1.13, 1.31) | <0.0001 | 1.43 (1.29, 1.60) | <0.0001 |

Values are odds ratios and 95% confidence intervals for BMI in 2015-17 (1 kg/m^2^ increments), centered at 25 kg/m^2^, controlling for: BMI latent class trajectory from infancy through mid-adulthood, age, birth village, current residence, SES, low physical activity, and smoking status in 2015-17. Confidence intervals account for clustering at the mother level.

1. Abdominal obesity defined as waist circumference >88 for women and >102 cm for men.
2. Modeled without smoking status due to non-convergence.
3. Obesity by percent body fat defined as body fat ≥32% for women and ≥25% for men.
4. Elevated triglycerides defined as ≥150 mg/dL or statin use.
5. Low HDL-c defined as HDL-c <50 mg/dL for women and <40 mg/dL for men.
6. Diabetes defined according to the American Diabetes Association diagnostic criteria: fasting plasma glucose ≥126 mg/dL, and/or post-challenge glucose ≥200 mg/dL, and/or diabetes medication use.
7. Hypertension defined according to the 2017 ACC/AHA/AAPA/ABC/ACPM/AGS/APhA/ASH/ASPC/NMA/PCNA Guideline for the Prevention, Detection, Evaluation, and Management of High Blood Pressure in Adults: systolic blood pressure ≥130 mmHg and/or diastolic blood pressure ≥90 mmHg and/or anti-hypertensive medication use.
8. Metabolic syndrome defined according to 2005 National Cholesterol Education Program Adult Treatment Panel III diagnostic criteria based on presence ≥3 of the following: abdominal obesity (waist circumference >88 for women and >102 cm for men); fasting plasma glucose ≥100 mg/dL or medication; triglycerides ≥150 mg/dL or medication; HDL-c <50 mg/dL for women and <40 mg/dL for men; and blood pressure ≥130 mmHg systolic, ≥85 mmHg diastolic and/or medication use.

Abbreviations: BMI, body mass index; HDL-c, high-density lipoprotein cholesterol; INCAP, Institute of Nutrition for Central America and Panama; SES, socioeconomic status.
