# Supplementary material for: Lifecourse body mass index trajectories and cardio-metabolic disease risk in Guatemalan adults
Source: PLoS One. 2020 Oct 22;15(10):e0240904. doi: 10.1371/journal.pone.0240904 (PMC7580923; doi:10.1371/journal.pone.0240904)
Supplement: S2 Table — (DOCX) [file pone.0240904.s002.docx]

S2 Table. Multivariable Logistic Regression Models to Predict Cardio-metabolic Risk Factors in 2015-17 After 40 Years of Follow Up at Age 37-54y Based on Weight-for-height Z Score (WHZ) at Age 18-42 Months in the INCAP Nutrition Supplementation Trial Longitudinal Cohort (n=387 Women, n=281 Men).

|  | Women  WHZ at 18-42 months  (1-unit increments) | | Men  WHZ at 18-42 months  (1-unit increments) | |
| --- | --- | --- | --- | --- |
| Cardio-metabolic risk factor | OR (95% CI) | *P* | OR (95% CI) | *P* |
| Obesity defined by BMI^a^ |  |  |  |  |
| Model 1 | 1.21 (0.92, 1.58) | 0.2 | 1.88 (1.35, 2.63) | 0.0002 |
| Model 2 | 1.22 (0.92, 1.60) | 0.2 | 1.82 (1.28, 2.59) | 0.0009 |
| Model 3 | - | - | - | - |
| Abdominal obesity defined by waist circumference^b^ |  |  |  |  |
| Model 1 | 1.37 (0.99, 1.88) | 0.05 | 1.81 (1.30, 2.53) | 0.0005 |
| Model 2 | 1.40 (0.99, 1.98) ^c^ | 0.05 | 1.78 (1.27, 2.51) | 0.0009 |
| Model 3 | 1.23 (0.69, 2.18) ^c^ | 0.5 | 0.86 (0.45, 1.65) | 0.6 |
| Obesity defined by % body fat^d^ |  |  |  |  |
| Model 1 | ^e^ | - | 1.26 (0.87, 1.81) | 0.2 |
| Model 2 | ^e^ | - | 1.19 (0.82, 1.71) | 0.3 |
| Model 3 | ^e^ | - | 0.72 (0.48, 1.08) | 0.1 |
| Elevated triglycerides^f^ |  |  |  |  |
| Model 1 | 1.11 (0.84, 1.46) | 0.5 | 1.47 (1.04, 2.08) | 0.03 |
| Model 2 | 1.11 (0.84, 1.48) | 0.4 | 1.35 (0.95, 1.90) | 0.09 |
| Model 3 | 1.06 (0.79, 1.42) | 0.7 | 1.08 (0.75, 1.55) | 0.7 |
| Low HDL-c^g^ |  |  |  |  |
| Model 1 | 1.04 (0.72, 1.51) | 0.8 | 1.67 (1.12, 2.49) | 0.01 |
| Model 2 | 1.11 (0.78, 1.58) ^c^ | 0.6 | 1.56 (1.03, 2.37) | 0.03 |
| Model 3 | 1.04 (0.72, 1.49) ^c^ | 0.8 | 1.18 (0.76, 1.84) | 0.4 |
| Diabetes^h^ |  |  |  |  |
| Model 1 | 0.80 (0.56, 1.17) | 0.2 | 1.37 (0.91, 2.05) | 0.1 |
| Model 2 | 0.81 (0.55, 1.19) | 0.3 | 1.24 (0.79, 1.94) | 0.3 |
| Model 3 | 0.81 (0.55, 1.19) | 0.3 | 1.31 (0.81, 2.12) | 0.3 |
| Hypertension^i^ |  |  |  |  |
| Model 1 | 0.93 (0.72, 1.19) | 0.5 | 1.29 (0.97, 1.74) | 0.08 |
| Model 2 | 0.91 (0.71, 1.17) | 0.5 | 1.26 (0.93, 1.70) | 0.1 |
| Model 3 | 0.85 (0.66, 1.09) | 0.2 | 1.13 (0.81, 1.56) | 0.5 |
| Metabolic syndrome^j^ |  |  |  |  |
| Model 1 | 0.97 (0.74, 1.28) | 0.8 | 1.54 (1.15, 2.06) | 0.003 |
| Model 2 | 0.99 (0.74, 1.31) ^c^ | 0.9 | 1.49 (1.11, 2.00) | 0.009 |
| Model 3 | 0.85 (0.62, 1.16) ^c^ | 0.3 | 1.07 (0.77, 1.49) | 0.7 |

Sample sizes were 387 and 281 (obesity defined by BMI, abdominal obesity defined by waist circumference, hypertension), 381 and 268 (elevated triglycerides, low HDL-c, metabolic syndrome, diabetes), and 376 and 269 (obesity defined by percent body fat) for women and men, respectively. Values are odds ratios and 95% confidence intervals for weight-for-height z score (WHZ) at 18-42 months (1 SD increments) controlling for: age and birth village (Model 1); current residence, SES, low physical activity, and smoking status in 2015-17 (Model 2); and BMI in 2015-17 (Model 3). Confidence intervals account for clustering at the mother level.

1. Obesity by BMI defined as BMI ≥30 kg/m^2^.
2. Abdominal obesity defined as waist circumference >88 for women and >102 cm for men.
3. Modeled without smoking status due to non-convergence.
4. Obesity by percent body fat defined as body fat ≥32% for women and ≥25% for men.
5. Non-positive definite solution.
6. Elevated triglycerides defined as ≥150 mg/dL or medication.
7. Low HDL-c defined as HDL-c <50 mg/dL for women and <40 mg/dL for men.
8. Diabetes defined according to the American Diabetes Association diagnostic criteria: fasting plasma glucose ≥126 mg/dL, and/or post-challenge glucose ≥200 mg/dL, and/or diabetes medication use.
9. Hypertension defined according to the 2017 ACC/AHA/AAPA/ABC/ACPM/AGS/APhA/ASH/ASPC/NMA/PCNA Guideline for the Prevention, Detection, Evaluation, and Management of High Blood Pressure in Adults: systolic blood pressure ≥130 mmHg and/or diastolic blood pressure ≥90 mmHg and/or anti-hypertensive medication use.
10. Metabolic syndrome defined according to the American Heart Association/National Heart, Lung, and Blood Institute scientific statement diagnostic criteria based on presence ≥3 of the following: abdominal obesity (waist circumference >88 cm for women and >102 cm for men); fasting plasma glucose ≥100 mg/dL or medication; triglycerides ≥150 mg/dL or medication; HDL-c <50 mg/dL for women and <40 mg/dL for men; and blood pressure ≥130 mmHg systolic, ≥85 mmHg diastolic and/or medication use.

Abbreviations: BMI, body mass index; HDL-c, high density lipoprotein cholesterol; INCAP, Institute of Nutrition for Central America and Panama; SES, socioeconomic status.
