# Supplementary material for: Lifecourse body mass index trajectories and cardio-metabolic disease risk in Guatemalan adults
Source: PLoS One. 2020 Oct 22;15(10):e0240904. doi: 10.1371/journal.pone.0240904 (PMC7580923; doi:10.1371/journal.pone.0240904)
Supplement: S3 Table — (DOCX) [file pone.0240904.s003.docx]

S3 Table. Multivariable Logistic Regression Models to Predict Cardio-metabolic Risk Factors in 2015-17 After 40 Years of Follow Up at Age 37-54 Years Based on BMI in 1988-89 in the INCAP Nutrition Supplementation Trial Longitudinal Cohort (n=460 Women, n=292 Men).

|  | Women | | Men | |
| --- | --- | --- | --- | --- |
|  | BMI in 1988-89  (1 kg/m^2^ units),  centered at 25 kg/m^2^ | | BMI in 1988-89  (1 kg/m^2^ units),  centered at 25 kg/m^2^ | |
| Cardio-metabolic risk factor | OR (95% CI) | *P* | OR (95% CI) | *P* |
| Obesity defined by BMI^a^ |  |  |  |  |
| Model 1 | 1.38 (1.27, 1.50) | <0.0001 | 1.50 (1.26, 1.79) | <0.0001 |
| Model 2 | 1.39 (1.27, 1.51) | <0.0001 | 1.51 (1.26, 1.82) | <0.0001 |
| Model 3 | - | - | - | - |
| Abdominal obesity defined by waist circumference^b^ |  |  |  |  |
| Model 1 | 1.30 (1.14, 1.47) | <0.0001 | 1.54 (1.29, 1.82) | <0.0001 |
| Model 2 | 1.31 (1.16, 1.49) ^c^ | <0.0001 | 1.53 (1.29, 1.83) | <0.0001 |
| Model 3 | 0.99 (0.80, 1.22) ^c^ | 0.9 | 1.24 (0.73, 2.10) | 0.4 |
| Obesity defined by % body fat^d^ |  |  |  |  |
| Model 1 | 1.20 (0.95, 1.50) | 0.1 | 1.16 (0.95, 1.40) | 0.1 |
| Model 2 | 1.19 (0.93, 1.52) ^c^ | 0.2 | 1.15 (0.95, 1.39) | 0.1 |
| Model 3 | 0.89 (0.63, 1.24) ^c^ | 0.5 | 0.81 (0.63, 1.03) | 0.08 |
| Elevated triglycerides^e^ |  |  |  |  |
| Model 1 | 1.07 (0.98, 1.16) | 0.1 | 1.09 (0.92, 1.29) | 0.3 |
| Model 2 | 1.07 (0.98, 1.16) | 0.1 | 1.08 (0.90, 1.28) | 0.4 |
| Model 3 | 1.01 (0.92, 1.11) | 0.8 | 0.87 (0.70, 1.07) | 0.2 |
| Low HDL-c^f^ |  |  |  |  |
| Model 1 | 1.13 (1.01, 1.27) | 0.04 | 1.18 (0.99, 1.40) | 0.06 |
| Model 2 | 1.13 (1.00, 1.27) ^c^ | 0.04 | 1.18 (1.00, 1.40) | 0.05 |
| Model 3 | 1.04 (0.91, 1.18) ^c^ | 0.6 | 0.95 (0.77, 1.17) | 0.6 |
| Diabetes^g^ |  |  |  |  |
| Model 1 | 1.21 (1.10, 1.33) | <0.0001 | 1.34 (1.07, 1.69) | 0.01 |
| Model 2 | 1.20 (1.10, 1.32) | <0.0001 | 1.40 (1.12, 1.74) | 0.003 |
| Model 3 | 1.29 (1.17, 1.44) | <0.0001 | 1.43 (1.11, 1.84) | 0.005 |
| Hypertension^h^ |  |  |  |  |
| Model 1 | 1.10 (1.02, 1.17) | 0.008 | 1.20 (1.05, 1.37) | 0.007 |
| Model 2 | 1.11 (1.03, 1.19) | 0.004 | 1.20 (1.05, 1.37) | 0.009 |
| Model 3 | 1.04 (0.96, 1.12) | 0.3 | 1.07 (0.92, 1.25) | 0.3 |
| Metabolic syndrome^i^ |  |  |  |  |
| Model 1 | 1.13 (1.03, 1.24) | 0.008 | 1.25 (1.09, 1.44) | 0.002 |
| Model 2 | 1.13 (1.03, 1.24) | 0.01 | 1.25 (1.09, 1.45) | 0.002 |
| Model 3 | 1.01 (0.90, 1.11) | 0.9 | 0.96 (0.80, 1.15) | 0.7 |

Sample sizes were 460 and 292 (obesity defined by BMI, abdominal obesity defined by waist circumference, hypertension), 453 and 285 (elevated triglycerides, low HDL-c, metabolic syndrome), 454 and 285 (diabetes), and 447 and 282 (obesity defined by percent body fat) for women and men, respectively. Values are odds ratios and 95% confidence intervals for BMI in 1988-89 (1 kg/m^2^ increments), centered at 25 kg/m^2^, controlling for: age and birth village (Model 1); current residence, SES, low physical activity, and smoking status in 2015-17 (Model 2); and BMI in 2015-17 (Model 3). Confidence intervals account for clustering at the mother level.

1. Obesity by BMI defined as BMI ≥30 kg/m^2^.
2. Abdominal obesity defined as waist circumference >88 for women and >102 cm for men.
3. Modeled without smoking status due to non-convergence.
4. Obesity by percent body fat defined as body fat ≥32% for women and ≥25% for men.
5. Elevated triglycerides defined as ≥150 mg/dL or medication.
6. Low HDL-c defined as HDL-c <50 mg/dL for women and <40 mg/dL for men.
7. Diabetes defined according to the American Diabetes Association diagnostic criteria: fasting plasma glucose ≥126 mg/dL, and/or post-challenge glucose ≥200 mg/dL, and/or diabetes medication use.
8. Hypertension defined according to the 2017 ACC/AHA/AAPA/ABC/ACPM/AGS/APhA/ASH/ASPC/NMA/PCNA Guideline for the Prevention, Detection, Evaluation, and Management of High Blood Pressure in Adults: systolic blood pressure ≥130 mmHg and/or diastolic blood pressure ≥90 mmHg and/or anti-hypertensive medication use.
9. Metabolic syndrome defined according to the American Heart Association/National Heart, Lung, and Blood Institute scientific statement diagnostic criteria based on presence ≥3 of the following: abdominal obesity (waist circumference >88 cm for women and >102 cm for men); fasting plasma glucose ≥100 mg/dL or medication; triglycerides ≥150 mg/dL or medication; HDL-c <50 mg/dL for women and <40 mg/dL for men; and blood pressure ≥130 mmHg systolic, ≥85 mmHg diastolic and/or medication use.

Abbreviations: BMI, body mass index; HDL-c, high density lipoprotein cholesterol; INCAP, Institute of Nutrition for Central America and Panama; SES, socioeconomic status.
