# Supplementary material for: Lifecourse body mass index trajectories and cardio-metabolic disease risk in Guatemalan adults
Source: PLoS One. 2020 Oct 22;15(10):e0240904. doi: 10.1371/journal.pone.0240904 (PMC7580923; doi:10.1371/journal.pone.0240904)
Supplement: S4 Table — (DOCX) [file pone.0240904.s004.docx]

S4 Table. Classifications of Obesity defined by Percent Body Fat, Waist Circumference, and Waist-height Ratio in 2015-17 After 40 years of Follow Up at Age 37-54 Years by BMI and Sex in the INCAP Nutrition Supplementation Trial Longitudinal Cohort (n=510 Women, n=346 Men).

|  | Women | | Men | |
| --- | --- | --- | --- | --- |
| Classification of Obesity | No Obesity  (BMI <30 kg/m^2^)  % (n) | Obesity  (BMI ≥30 kg/m^2^)  % (n) | No Obesity  (BMI <30 kg/m^2^)  % (n) | Obesity  (BMI ≥30 kg/m^2^)  % (n) |
| Obesity defined by percent body fat^1^ |  |  |  |  |
| No | 5.8 (17) | 0.0 (0) | 28.5 (77) | 4.8 (3) |
| Yes | 94.2 (278) | 100.0 (202) | 71.5 (193) | 95.2 (59) |
| Abdominal obesity defined by waist circumference^2^ |  |  |  |  |
| No | 16.5 (50) | 0.0 (0) | 95.0 (268) | 9.4 (6) |
| Yes | 83.5 (254) | 100.0 (206) | 5.0 (14) | 90.6 (58) |
| Abdominal obesity defined by waist-height ratio^3^ |  |  |  |  |
| No | 0.7 (2) | 0.0 (0) | 15.6 (44) | 0.0 (0) |
| Yes | 99.3 (302) | 100.0 (206) | 84.4 (238) | 100.0 (64) |

Values presented are percentages.

1. Obesity by percent body fat defined as body fat ≥32% for women and ≥25% for men.
2. Abdominal obesity defined as waist circumference >88 cm for women and >102 cm for men.
3. Abdominal obesity by waist-height ratio defined as waist-height ratio >0.50.

Abbreviations: BMI, body mass index; INCAP, Institute of Nutrition for Central America and Panama.
