# Supplementary material for: Lifecourse body mass index trajectories and cardio-metabolic disease risk in Guatemalan adults
Source: PLoS One. 2020 Oct 22;15(10):e0240904. doi: 10.1371/journal.pone.0240904 (PMC7580923; doi:10.1371/journal.pone.0240904)
Supplement: Attachment — Submitted filename: Response letter to PLOS ONE 14 JAN_v2.docx [file pone.0240904.s005.docx]

January 14, 2020

Dear Editor,

We are writing concerning the decision that the Research Article “Lifecourse body mass index trajectories and cardio-metabolic disease risk in Guatemalan adults” was too closely related to an article published in 2017 using data from the same cohort.

The Research Article we submitted for your consideration used data from the INCAP Nutrition Supplementation Trial Longitudinal Cohort cohort to derive lifecourse body mass index (BMI) trajectories using up to 22 height and weight measurements collected from infancy through mid-adulthood (1969-2004). We examined whether the lifecourse BMI trajectories were associated with cardiometabolic disease in the 2015-16 follow-up study wave.

The article referenced in the decision letter (Ford ND, Jaacks LM, Martorell R, Mehta NK, Perrine CG, Ramirez-Zea M, Stein AD. Dietary patterns and cardio-metabolic risk in a population of Guatemalan young adults. BMC nutrition. 2017 Dec;3(1):68) used data from the 2002-04 follow-up study of the INCAP cohort to derive dietary patterns using principal components analysis. We then explored whether the dietary patterns were associated with cardio-metabolic disease outcomes in 2002-04.

Although both articles use data from the INCAP Cohort and examine associations between exposures and cardiometabolic disease outcomes, both the exposures (lifecourse BMI trajectories vs. dietary patterns) and the years in which the cardiometabolic disease outcomes were measured (2015-16 vs. 2002-04) in the two articles differ.

We respectfully request that our article is reconsidered by the editorial board for review. The two papers are distinct enough with respect to data and focus that we do not feel that citing the 2017 in the present paper is merited.

Thank you for your consideration.

Best,

Aryeh D. Stein, PhD (*corresponding author*)

Hubert Department of Global Health, Emory University, 1518 Clifton Road NE, CNR 7007, Atlanta, GA, USA

Email: [aryeh.stein@emory.edu](mailto:aryeh.stein@emory.edu)

Phone: 404-727-4255

Nicole D. Ford, PhD, MPH

Hubert Department of Global Health, Emory University, Atlanta, GA, USA

Reynaldo Martorell, PhD

Hubert Department of Global Health, Emory University, Atlanta, GA, USA

Neil K. Mehta, PhD, MA, MSc

Department of Health Management and Policy, School of Public Health, University of Michigan, Ann Arbor, MI, USA

Cria G. Perrine, PhD, MA

Division of Nutrition, Physical Activity, and Obesity, U.S. Centers for Disease Control and Prevention, Atlanta, GA, USA

Manuel Ramirez-Zea, MD, PhD

INCAP Research Center for the Prevention of Chronic Diseases (CIIPEC), Institute of Nutrition of Central America and Panama, Guatemala City, Guatemala
