# Supplementary material for: Lifecourse body mass index trajectories and cardio-metabolic disease risk in Guatemalan adults
Source: PLoS One. 2020 Oct 22;15(10):e0240904. doi: 10.1371/journal.pone.0240904 (PMC7580923; doi:10.1371/journal.pone.0240904)
Supplement: Attachment — Submitted filename: Responses to Reviewers_11 AUG.docx [file pone.0240904.s006.docx]

**Responses to Reviewers**

| **Reviewer 1** | |
| --- | --- |
| This is a well written manuscript, but there are some weaknesses in the analytical approach and discussion of the results. The paper is not giving entirely novel information, but the authors may be able to strengthen existing knowledge by expanding the paper with more detailed discussion of results. Especially, the rather one-sided sample composition with a very high rate of overweight and obese participants lacks appropriate discussion and meaningful conclusions. | Thank you for your valuable feedback. We have made substantial changes to the methods and discussion sections to address the gaps you identified in your review. |
| There are some inconsistencies in spacing and formatting:  - l.168: “≥ 3 days of vigorous activity of ≥20 min/day” | Corrected. |
| - l.54 and l.64: inconsistent use of hyphen: “middle-income country” and “high income country” | Corrected. |
| - l.70-71: inconsistent use of dash and hyphen: “early life – especially (…) economies - have”, or l.68: (16-18) vs. l.69 (19–22). | Corrected. |
| Abstract:  - l.25: Please give the total N of study participants | We added the total number of study participants.  “We used logistic regression to assess the role of BMI trajectory on CMD risk in 510 women and 346 men (N=856).” |
| - Please clarify that the three latent classes for men were collapsed to two classes for analysis. | “Given the small percentage of men in the high BMI latent class, we collapsed the medium and high BMI latent classes for men (*n* = 205, 59.1%).” |
| Methods: - l.82-84: What kind of nutritional intervention and at what age where infant recruited? Did the intervention influence any of the results/adjustment for intervention groups needed? What was the total n of participants in the study? | Thank you for this feedback. We’ve added the following to the description of the intervention.  “The original community-randomized intervention trial was designed to assess the influence of improved early life nutrition on cognitive and physical development (N=2,392). Two sets of matched villages were randomized to *Atole*, a protein-energy nutritional supplement, or *Fresco*, a low-energy beverage made from sugar and water. Children could be exposed prenatally through maternal consumption as well as postnatally through breastmilk or the child’s own supplement intake. Exposure to *Atole* before age 3 years was positively with weight and linear growth and negatively associated with fat-folds, suggesting that increases in weight were largely due to fat-free mass (28).”  *Reference*  Hoddinott J, Behrman JR, Maluccio JA, Melgar P, Quisumbing AR, Ramirez-Zea M, Stein AD, Yount KM, Martorell R. Adult consequences of growth failure in early childhood. The American journal of clinical nutrition. 2013 Nov 1;98(5):1170-8. |
| - l.90-92: Please provide details when informed consent was given by whom. As participants were recruited already in infancy, initial consent was given by the parents? Was an informed consent obtained from participants themselves at a later follow-up? | We updated the text to clarify that caregivers provided consent on behalf of their children during the study waves where the children were minors.  “Caregivers consented on behalf of their children in the original trial (1969-77) and in the first follow-up wave (1988-89) if the participants were minors. For all other follow-up waves, participants gave written informed consent.” |
| - Sentence in l. 99 to 101 is missing a verb: …trajectories were ?derived/estimated? from… | Thank you. We corrected this error.  “Using LCGA, sex-specific BMI latent class trajectories were derived from…” |
| - Please revise the order of sub-headings/-sections of this section, e.g. the description of BMI trajectories should come after the description of measurements and probably after the statistical analysis section. Adding the trajectories part to the statistical analysis section is also possible | Thank you for this feedback. We moved the section on deriving the BMI trajectories after the description of the data collection. |
| - There should be more information on actual numbers of BMI values involved in LCGA and a more detailed method description making the manuscript more readable without ref. 24. | We added more details to this section, including the number of BMI values used, the number of classes examined, and fit statistics for the selected models. |
| - There is no actual reason to summarize the medium and high class from the LCGA modelling in men into one high class. To distinguish between severely obese and others would make sense. | Due to the small sample size of men in the highest class (n=45) and the distributions of the covariates and outcomes, the multi-variable models using 3 BMI trajectory classes for men would not converge. We realize that the rationale for collapsing these categories was not clear in the original manuscript. We updated the paragraph describing the trajectory classes to include this information. |
| - Please add a definition of all parameters included in the definition used for the CMD risk profiles, before the description of methods used to measure these parameters | We added a short paragraph defining all the outcomes.  Lines 148-153  “For CMD risk profiles in 2016, we assessed two measures of overall adiposity – BMI category and percent body fat – and two measures of central adiposity – abdominal obesity defined by waist circumference and waist-height ratio (WHtR). We additionally evaluated blood lipids, fasting blood glucose, 2-hour post-challenge glucose, blood pressure, and metabolic syndrome. Metabolic syndrome (MetS) is a group of interrelated metabolic risk factors that increase risk of conditions such as cardiovascular disease, stroke, and diabetes (31).” |
| - Same for the MetS: a short rationale would be helpful, as this syndrome is first mentioned in the methods section without context | We added a definition of MetS. Please see response to the comment above about definitions of the outcomes. |
| - l.148: Change defines to defined | Corrected. |
| - l. 162: Please give a short rationale or reference for this dichotomization | Other than Guatemala City, all other residences in the study population were either rural or semi-rural.  “Current residence was classified as Guatemala City vs. other (rural and semi-rural).” |
| - l.162: Was parity included in all models? Please specify | We did not include parity in the final adjusted models. We identified potential covariates based on their theoretical relationship with the CMD risk factors. Among these factors, we selected the final covariates for the models by assessing bivariate relationships between the potential covariates and the outcomes. Although we assessed parity, multivitamin, and alcohol use, these were not associated with the outcomes and were thus not included in the final adjusted models. We added the following statements in the methods section to clarify:  Lines 227-229:  “We identified the following potential covariates based on their theoretical relationship with the CMD risk outcomes including, socioeconomic status (SES), current residence, parity, smoking status, current vitamin intake, current alcohol consumption, and physical activity level.”  Lines 313-315:  “Among the potential covariates, we selected the final covariates by assessing bivariate relationships between the potential covariates and the outcomes. Parity, multivitamin intake, and alcohol consumption were not included in the final adjusted models.” |
| - l. 177-186: more fitting into the results section (together with Fig 2) | Thank you for your feedback. We prefer to keep the description of the analytic sample with the methods for secondary data analyses. |
| - l. 164: In what model were these two variables used? Can’t find them in the statistical analysis section. Please also provide a short rationale/reference on why to include them into the analysis | We did not include either of these variables in the final adjusted models. Please see the response above to the comment above about parity. |
| - l.201-202: What about the other lifestyle covariates (parity, multivitamin and alcohol use)? How were the covariates selected for the model? | We identified potential covariates based on their theoretical relationship with the CMD risk factors. Among these factors, we selected the final covariates for the models by assessing bivariate relationships between the potential covariates and the outcomes. Although we assessed parity, multivitamin, and alcohol use, these were not associated with the outcomes and were thus not included in the final adjusted models. We added the following statements in the methods section to clarify.  Lines 227-229:  “We identified the following potential covariates based on their theoretical relationship with the CMD risk outcomes including, socioeconomic status (SES), current residence, parity, smoking status, current vitamin intake, current alcohol consumption, and physical activity level.”  Lines 313-315:  “Among the potential covariates, we selected the final covariates by assessing bivariate relationships between the potential covariates and the outcomes. Parity, multivitamin intake, and alcohol consumption were not included in the final adjusted models.” |
| - Please clarify for each model what is the exact outcome and what is the exact main predictor. | As described, the main predictor in these analyses is BMI latent class. Lines 315-320 lists all outcomes. |
| - A sensitivity analysis including all three latent classes for men might give some interesting results (depending on the size of the groups and outcome-diversity within these groups) | As described above in response to the rationale behind collapsing the medium and high categories in men, the multi-variable models using 3 BMI trajectory classes for men would not converge. |
| - Did you check for direct associations of BMI with different age? This might be more interesting than the usage of BMI classes. | Yes. In sensitivity analyses, we evaluated the association between CMD risk factors in 2016 with: (1) a single measure of adiposity in childhood (weight-for-height Z score [WHZ] at age 18-42 months); and (2) a measure of adiposity in adolescence/young adulthood (BMI in 1988-89 at age 10-27 years) using the same modeling strategy as the primary analyses. The results are presented in the manuscript and in Supplemental Tables S2 and S3. Higher WHZ and 1988-89 BMI were generally associated with increased risk of CMD risk in 2016; however, apart from diabetes, these associations were not significant after adjusting for current BMI. Our findings were robust in models using WHZ from 18-42 months and BMI in 1988-89 as the primary exposures in sensitivity analyses, likely because BMI tracked over time. |
| Results: - l. 256-261: are these numbers normal? They appear very high to me. Possibly discuss this in the discussion section | This level of obesity is not uncommon in Latin America – particularly among women. Latin America, the Pacific Islands, and the Middle East regions have the highest burden of overweight and obesity. According to the Global Burden of Disease study, more than 20% of women aged 15 years and older in Latin America were obese (defined by BMI) as of 2013. Higher obesity prevalence in our sample compared to the GBD study is not surprising considering the participants are older (37-54 years). Other measures of obesity, such as waist circumference, waist-height ratio, and percent body fat are infrequently measured in large-scale population-based surveys.  *References*  Ford ND, Patel SA, Narayan KV. Obesity in low-and middle-income countries: burden, drivers, and emerging challenges. Annual review of public health. 2017 Mar 20;38:145-64.  Ng M, Fleming T, Robinson M, Thomson B, Graetz N, Margono C, Mullany EC, Biryukov S, Abbafati C, Abera SF, Abraham JP. Global, regional, and national prevalence of overweight and obesity in children and adults during 1980–2013: a systematic analysis for the Global Burden of Disease Study 2013. The lancet. 2014 Aug 30;384(9945):766-81. |
| - l. 269: Clarify: obesity at what age/time point; obesity by BMI? What is the reason to regress Obesity defined by BMI on a BMI trajectory class. | As described in the methods section, all outcomes in the paper were measured in 2015-17. The BMI trajectories were developed using length/height and weight measurements from 1969-2004. The 12-year lag between the final weight/height in the BMI latent class trajectory and the CMD risk factors was implemented to control for potential reverse causation between adult BMI and CMD risk. |
| Discussion:  - l. 329-334 and l.348-349: this indicates that the composition of this sample might not be optimal to answer the research question asked in the beginning of the manuscript. Please elaborate this aspect more | Although this population may not be optimal to answer the research question due to high levels of overweight and obesity, there are no alternative data sources. To our knowledge, the INCAP longitudinal cohort is the only study population with length/height and weight measurements from infancy through mid-adulthood in a LMIC. In longstanding cohort studies from high-income countries, participants have not been exposed to early life undernutrition coupled with later life obesogenic environments. Among cohort studies from LMIC, data are available for more limited segments of the lifecourse (e.g. the Birth to Twenty cohort from South Africa) due to the relatively younger age of the study participants compared to the INCAP longitudinal cohort.  Lines 522-527:  “Considering BMI > 23 kg/m^2^ increases risk of CMD (51) and neither of the women’s BMI latent class trajectories had a median BMI in the normal range, high levels of and lack of variation in adiposity in women could explain our inability to detect associations between BMI latent class trajectory and CMD risk factors. However, to our knowledge, the INCAP longitudinal cohort is the only study population available to answer our research question, owing to the relatively young age of the study participants in other LMIC cohorts.” |
| - l. l. 334: why? This conclusion seems kind of far fetched as most of the associations of BMI with CMD factors are no influence by the “BMI throughout life” i.e. the trajectories in this case but the present BMI. | We edited the sentence to clarify.  “Because BMI tracked from early childhood into adulthood and current BMI was strongly associated with CMD risk factors, our findings highlight the importance of keeping a healthy BMI throughout life.”  Although the BMI trajectories were not associated with CMD risk factor outcomes among women and were mediated by current BMI among men, our sensitivity analyses support our conclusion that supporting healthy growth in early life and preventing weight gain in adulthood are important. Higher adiposity in childhood (WHZ at age 18-42 mo) and in adolescence/young adulthood (BMI in 1988-89 at age 10-27 y) were both generally associated with increased odds of CMD risk factors in 2016. |
| - l. 337: Please define “large” differences in BMI trajectories. | We presented the differences in BMI by trajectory class in the results section:  “In 2016, the difference in mean BMI between the low and high/medium BMI latent class trajectories was 2.0 kg/m^2^ for women and 1.8 kg/m^2^ for men.”  However, we removed “large” from this sentence in the discussion. |
| - l.338: Why “pre-conceptual factors”? I think it is not possible to draw conclusions about this period based on the results. | Pre-conceptual and gestational factors shape birth size and early infant weight gain. Some of these characteristics include paternal stature, parity, pre-pregnancy BMI, gestational weight gain, gestational diabetes, hypertension, infection, anemia, among others.  *References*  Hindmarsh PC, Geary MP, Rodeck CH, Kingdom JC, Cole TJ. Factors predicting ante-and postnatal growth. Pediatric research. 2008 Jan;63(1):99-102. |
| - l. 341-349: Please clarify what your study adds to the current knowledge. The first and last sentence of this paragraph have no connection for me | We updated this paragraph to more clearly state the contribution of our study to current knowledge. |
| - l. 350-351: in this study or in general? | We updated this sentence.  “High levels of adiposity in adulthood might explain the lack of association between BMI latent class trajectory class and CMD risk in women in this study.” |
| - l. 350-362: in this paragraph you greatly highly the shortfalls of the sample composition in relation to the research question. Please elaborate, what insights you did get despite this unfavourable sample composition. | As described above, there are no alternative data sources to address the research question. To our knowledge, the INCAP longitudinal cohort is the only study population with length/height and weight measurements from infancy through mid-adulthood in a LMIC. |
| - l. 377-378: Measurements to build such trajectories of waist circumference or body fat were not available in your study? Or what is the reason to only built BMI trajectories in this cohort | Waist circumference and percent body fat are not available in all study waves. LCGA requires a minimum of 3 measurements to develop trajectories. Additional data points allow for more stable trajectory models. Ninety-five percent of the participants included in the trajectory development had more than 3 BMI values.  Further, because the methods used to measure percent body fat differ by study wave (e.g. calipers, underwater hydrostatic weighing, and deuterium oxide dilution technique), it may not be advisable to compare across time.  *Reference*  Jung T, Wickrama KA. An introduction to latent class growth analysis and growth mixture modeling. Social and personality psychology compass. 2008 Jan;2(1):302-17. |
| - l.378: Please discuss more the role of early life undernutrition in your results | We added more information about the role of undernutrition, including the findings of a paper evaluating the role of nutrition supplementation on BMI trajectory class.  Lines 469-472  “Nutrition supplementation from conception to age 2 years was not associated with BMI latent class membership in this cohort; however, higher childhood socioeconomic status was associated with increased odds of high BMI latent class membership in both men and women (43).”  Lines 474-486  “Some study participants were exposed to a nutrition supplement that improved child growth, 86% of the cohort was stunted at age 2 years (HAZ <-2 SD), suggesting severe early life undernutrition even with the intervention.” |
| - l. 400: “and preventing weight gain in adulthood” I don’t think your research support this conclusion. Please rewrite the conclusion with more relation to the initial research question you formulated | We respectfully disagree with the reviewer on this point. Model 3 formally evaluated the mediating role of current BMI on CMD risk factors. The results are presented in Supplemental Table 1. Except for diabetes, current BMI was strongly associated with all CMD risk factors in our study, irrespective of BMI trajectory from infancy through mid-adulthood.  We updated the conclusion to clarify and also provided more information in the discussion to support this conclusion.  Lines 463-465:  “Because BMI tracked from early childhood into adulthood and current BMI was strongly associated with CMD risk factors, our findings highlight the importance of keeping a healthy BMI throughout life.”  Lines 489-495:  “Even though the BMI latent classes were not clinically distinct with respect to CMD risk factors after adjusting for current BMI, our study has potentially important implications for prevention of chronic disease risk in contexts with early life undernutrition and later life caloric excess. Irrespective of BMI from childhood through mid-adulthood, current BMI was strongly associated CMD risk factor odds in this population, except for diabetes. Thus, maintaining healthy weight throughout the life course, including averting weight gain in mid-life, could be important in mitigating adult CMD risk.” |
| **Reviewer 2** | |
| In this report, the authors evaluated the association of latent class trajectories of BMI from infancy to middle adulthood with cardiometabolic disease risk factors in a retrospective analysis of a cohort of participants in Guatemala enrolled in 1962-77 and followed through 2015-17. No associations between BMI latent class and cardiometabolic disease risk factors were found after adjustment for current BMI in men, and an association was found only between BMI latent class and obesity by BMI in women. |  |
| 1. Introduction: Greater contextualization of cardiometabolic disease in Latin America is suggested. It would be helpful to know where in the epidemiologic transition Guatemala lies. E.g., lines 45-46: what is the quantitative proportion of CMD disease burden in Latin America? Line 50: What is the prevalence of obesity in children and adults in Guatemala? | Thank you for this feedback. We have added the prevalence of overweight and obesity, high fasting blood glucose, and high blood pressure among adults in Guatemala. We have also included the prevalence of overweight/obesity among children under 5 years. |
| 2. Introduction: A stronger narrative describing the connection between undernutrition and adult CMD is suggested. If “early life undernutrition is thought to increase risk of adult cardio-metabolic disease – particularly among people later exposed to hyper-caloric environments” (lines 52-53), is it the hyper-caloric environment that is the culprit? Additionally, more exposition on the relationship between childhood undernutrition and BMI trajectories would help support the rationale for this study. | We added a more detailed description of the links between early life undernutrition, obesogenic adult environments, and CMD risk factors.  “Early life undernutrition is thought to increase risk of adult cardio-metabolic disease (CMD) (6). Birth size has been consistently associated with adiposity, obesity, type 2 diabetes, lipid profile, and blood pressure later in childhood, adolescence, or adulthood (7-11). The Developmental Origins of Health and Disease Hypothesis posits that structural and functional adaptations to early life undernutrition might preserve brain and vital organ development in contexts of nutrient deprivation; however, these adaptations are a “mismatch” for later obesogenic environments in countries undergoing nutrition transition (6). Yet there is little data from low- and middle-income country (LMIC) populations about the long term consequences of persistent early life undernutrition in obesogenic environments (12). |
| 3. Introduction, paragraph 3 (lines 56-63): For a reader who may be less familiar with methods, it would be helpful to more explicitly state why the reasons stated in this paragraph make understanding the role of younger-age body size on adult disease more challenging. | Thank you for the comment. We have reorganized the introduction to make this point more clearly. |
| 4. Introduction, paragraph 3 (lines 56-63): A statement establishing a stronger connection between the first sentence (starting line 56) and this second one (starting line 60) is needed: in what way does LCGA help understand the role of childhood body size on adult disease risk? The connection between the two points is not clear. | We reorganized the introduction to make this point more clearly.  “…(LCGA) allows us to identify distinct growth patterns in cohort sub-groups not readily identifiable using other modeling techniques and to minimize collinearity of repeated measures (26). If people exposed to low nutrient environments in early life – especially those later exposed to hyper-caloric environments consistent with developing economies – have growth patterns that confer distinct risk for CMD, life course growth analysis could help identify differential risk.” |
| 5. Methods: Can the authors provide a brief summary of the findings of the trial? It would be helpful context in this narrative to know if the intervention resulted in changes in physical development, and how (if at all) the supplementation changes the representativeness of this population to the remainder of the childhood population in Guatemala? | Thank you for your comment. We added a sentence to the description of the study population to highlight main findings of the trial.  “Exposure to *Atole* before age 3 years was positively with weight and linear growth and negatively associated with fat-folds, suggesting that increases in weight were largely due to fat-free mass (28).”  We also updated the study limitations to include the potentially limited generalizability of our findings.  “The generalizability of our findings may be limited to contexts of high childhood undernutrition with subsequent exposure to obesogenic adult environments.” |
| 6. Methods (lines 110-114): Visually (Figure 1), the latent class BMI trajectories within-sex appear follow the same pattern. The primary difference appears to be that BMI levels are higher in the ‘high’ or ‘high/medium’ classes. In the context of “high levels of adiposity in both trajectory classes” (line 330), what is the authors’ interpretation of the populations the latent classes represent? In addition, what is the authors’ assessment of how clinically different/distinguishable the latent class trajectories are? Do the latent class trajectories represent a different population? The clinical difference between the latent class trajectories are not clear but should be discussed. | Before developing the latent class trajectories, we did not expect that BMI would track from infancy through adulthood as it tends to do in high income countries. Data from LMIC have generally shown that growth failure in childhood is associated with increased adiposity and obesity in adulthood. Thus, we hypothesized that the children with the poorest early life growth would have higher BMI in adulthood than the children who had more normal growth as children. However, despite the intervention, most participants were stunted at age 2 years, suggesting that few children had “normal” childhood growth in this cohort.  While LCGA helps identify heterogeneity in body size over life course, the classes are not “real” but instead reflecting a continuum of growth in the population and should be considered a tool to help visualize variability within the global distribution of BMI gain. We agree that the primary difference in the different trajectory classes is the higher classes have higher BMI over the lifecourse. Additionally, the higher BMI classes appear to have an increasing rate of weight gain beginning in their 30s. Between ages 34 y and 40 y, women in the high BMI trajectory class gained an average of 2.69 kg/m^2^ relative to 0.2 kg/m^2^ in the low BMI trajectory class. Men in the medium/high BMI trajectory class gained an average of 2.72 kg/m^2^ between 34 and 40 y compared to 0.08 kg/m^2^ in the low BMI trajectory class.  Even though the BMI latent classes were not clinically distinct with respect to CMD risk factors after adjusting for current BMI, our study has potentially important implications for prevention of chronic disease risk in contexts with early life undernutrition and later life caloric excess. Irrespective of BMI from childhood through mid-adulthood, current BMI was strongly associated CMD risk factor odds in this population, except for diabetes. Thus, maintaining healthy weight throughout the life course, including averting weight gain in mid-life, could be important in mitigating adult CMD risk. Among men in our study, the apparent benefit of lower BMI from childhood to early adulthood with respect to lipid profile was negated by weight gain between 2004 and 2016.  *Reference*  Stein AD, Thompson AM, Waters A. Childhood growth and chronic disease: evidence from countries undergoing the nutrition transition. Matern Child Nutr. 2005 Jul;1(3):177–84. |
| 7. The authors state “Because BMI latent class trajectory class was not associated CMD risk after controlling for current BMI and given that early life BMI tracked into adulthood, our findings highlight the importance of healthy growth early in life and preventing weight gain in adulthood.” (Discussion, lines 398-400). Was a formal mediation analysis done? This conclusion commenting on how the data highlight the importance of “healthy growth early in life and preventing weight gain,” while consistent with a broad range of other studies, seems to reach beyond what the results of the observed associations between latent class BMI trajectories and CMD risk factors show. | The 12-year lag between when the final weight/height in the BMI latent class trajectory and the CMD risk factors attempted to control the influence of potential reverse causation between adult BMI and CMD risk factors. However, we also examined the potential mediating role of current BMI on CMD risk factors. To this end, we included an additional model for each outcome (Model 3) that additionally controlled for BMI in 2016 in all models except where obesity defined by BMI was the outcome. The results are presented in Supplemental Table 1. Current BMI was significantly associated with all outcomes except for diabetes. We updated the results section to clarify:  “In the mediation analyses, BMI in 2016 was strongly associated with all CMD risk factor outcomes in both sexes except for diabetes, (S1 Table).”  Although the BMI trajectories were not associated with CMD risk factor outcomes among women and were mediated by current BMI among men, our sensitivity analyses support our conclusion that supporting healthy growth in early life and preventing weight gain in adulthood are. Higher adiposity in childhood (WHZ at age 18-42 mo) and in adolescence/young adulthood (BMI in 1988-89 at age 10-27 y) were both generally associated with increased odds of CMD risk factors in 2016. |
| 8. Given the generally high level of adiposity in all participants, have the authors considered further examining the subset of participants (if any) who are “metabolically normal obese”? | We did not examine this potential subset of participants for this study; however, we agree that it would be an interesting topic for future research in this cohort. |
| 9. Minor comment: Though this may be semantic, there is a clinical difference between CMD, CMD risk factors (e.g. obesity, cholesterol levels), and CMD risk (e.g., in the context of risk estimation models like the pooled cohort equations that are widely used in clinical practice for primary prevention of cardiovascular disease). The independent variables in this analysis seems to be CMD risk factors, so the authors may consider using “CMD risk factors” rather than “CMD risk” throughout to describe their results. | Thank you for this comment. We agree and have updated “CMD risk” to “CMD risk factors” throughout the manuscript. |
