# Supplementary material for: Lifecourse body mass index trajectories and cardio-metabolic disease risk in Guatemalan adults
Source: PLoS One. 2020 Oct 22;15(10):e0240904. doi: 10.1371/journal.pone.0240904 (PMC7580923; doi:10.1371/journal.pone.0240904)
Supplement: Attachment — Submitted filename: Responses to Reviewers_23 SEPT.docx [file pone.0240904.s007.docx]

**Responses to Reviewers**

| **Editor** | |
| --- | --- |
| As mentioned in my previous response on this paper, I understand that the authors may not be able to make the data publicly available - but I cannot see any reason why this would not be possible with respect to the analysis code. This would be very much in line with reproducible and open science. | We have uploaded the code to OSF. Please see:  Ford N. SAS code for “Lifecourse body mass index trajectories and cardio-metabolic disease risk in Guatemalan adults.” [Internet]. OSF; 2020. Available from: osf.io/5cvw8 |
